# Building a Culture of Health and Well-Being at Merck

**DOI:** 10.1089/pop.2018.0116

**Published:** 2019-09-26

**Authors:** Cathryn E. Gunther, Virginia Peddicord, Joseph Kozlowski, Yi Li, Danielle Menture, Raymond Fabius, Sharon Glave Frazee, Peter J. Nigro

**Affiliations:** ^1^ Merck & Co., Inc., North Wales, Pennsylvania.; ^2^ AB3Health, Newtown Square, Pennsylvania.; ^3^ Department of Pediatric Medicine, Washington University at St. Louis, St. Louis, Missouri.

**Keywords:** culture of health, population health, employer, workforce health, wellness program

## Abstract

There is increasing evidence that a healthy and safe workforce can provide a competitive business advantage. This article shares the efforts and experience of a large global employer as it builds on existing corporate wellness and safety programs to develop a corporate culture of health and well-being. Starting with a comprehensive review of the current state of employee health and culture, a small team established the business case, aligned strategic partners, created an implementation plan, and engaged the C-Suite. The aim of this article is to provide a case study that others might use to design their blueprint, to gain awareness and to build a culture of health and well-being within their organization.

## Introduction

### What is a culture of health?

The classic definition of population health is “the distribution of health outcomes within a population, the health determinants that influence distribution and the polices and interventions that impact the determinants.”^1^ Population health is an approach that aims to materially improve the health of specific populations such as employees of an organization, members of a health plan, citizens of a community, or a nation's population. For employers, population health typically focuses on the employee population as well as spouses/domestic partners and dependents of employees.

Population health is best achieved in an environment that supports a culture of health. The Health Enhancement Resource Organization (HERO), together with the Robert Wood Johnson Foundation, defines a culture of health as “one in which individuals and social entities (eg, households, organizations, etc) are able to make healthy life choices within a larger social environment that values, provides, and promotes options that are capable of producing health and well-being for everyone regardless of background or environment. In short, the healthy choice becomes the valued and easy choice.”^2^

Stated more simply, workplaces with a culture of health and wellness surround employees with the environment, policies, and cues that support making healthy choices on both a conscious and unconscious basis.^3^ To be successful at building a culture of health, companies must build workforce health and safety into the mission, vision, and values of the organization.

### Benefits of a culture of health

Developing a culture of health and well-being is important for many reasons. In today's competitive marketplace, it is advantageous for employers to take a holistic approach to assessing and mitigating health risks in their workforce. More than 2 decades of research suggest the importance of health and productivity as a business strategy.^4^

Employers with higher corporate health assessment scores, a common way to quantify “cultures of health,’’ tend to have a lower health care cost trend, without the need to reduce benefit services or shift more costs to their employees.^5^ Given that health care costs represent a significant expenditure for most employers, reducing health care costs is a competitive advantage. In fact, Warren Buffett recently cited health care as the “real corporate tax” because of the rate of escalation over the years.^6^

A healthier workforce is also more productive. It is estimated that for every dollar saved in direct health care costs, employers receive an extra $2.30 in improved performance or productivity.^7,8^ When an employee is unhealthy, first they do not perform optimally at work (presenteeism); then the work is not being completed in a timely matter (delayed production). Unwell workers may then not show up for work (absence), and ultimately, perhaps are even lost from the workforce (disability). All these steps have real impacts on the performance of an organization.

Even more compelling, other studies have shown superior stock performance by organizations that achieve a culture of health, as measured by receipt of various health and safety awards such as the American College of Occupational and Environmental Medicine's (ACOEM) Corporate Health Achievement Award (CHAA),^9^ the C. Everett Koop award,^10^ and being recognized as a high-scoring HERO organization.^11^

These benefits do not mean that building a culture of health is simple or even common. In fact, fewer than half of American workers say that the climate in their organization supports employee well-being.^12^ It takes a concerted effort, patience enough to play the long game, and financial investment to create supportive and effective workplace cultures that support health and wellness. This article aims to share the experience of a large global employer to assist others in designing their blueprint to gain awareness and to build an organizational culture of health and well-being.

## Building a Culture of Health and Well-Being: The Merck & Co. Case Study

The specifics of building a culture of health and well-being are inherently personal to a given organization as they consider 4 basic elements: why, who, what, and how. In this case study, Merck & Co. illustrates how the study team considered and then applied these elements to develop the business case for a culture of health initiative. The intent is that others can learn from this process as they design their own blueprint toward better employee health and well-being.

### Why

Why is perhaps most important. Building a culture of health is a significant undertaking. Having a clear vision of why it is being done provides a compass during the inevitable challenges that come with large company initiatives. The study team started with the company's business mission to save and improve lives. A healthy workforce is needed to do the important work of saving and improving the lives of others. This extended to a commitment to helping employees be well and stay safe.

The team developed a clear vision to become a benchmark role model for employers interested in building a culture of well-being by demonstrating the implementation of an evidence-based approach. The aim of this approach is to achieve clear improvements in the health and well-being of the workforce. Specifically, the vision is to optimize a culture of health, wellness, and safety with measurable improvement in targeted areas, including the improvement of health status. Additionally, the team aspired to be an exemplar for other companies by sharing learnings, best practices, and evidence that support the hypothesis that good health is good business.

As is stated on the Merck website: “We believe there are many benefits to this approach. The health and well-being of our workforce have a direct link to optimal workforce performance. Whether the job is done at a work location or at home, sickness, injury and stress can affect a person's ability to perform and contribute effectively. Because our business is promoting optimal health, we believe we must lead by example. We also believe that a constructive approach to our employees' health and overall wellbeing, in all aspects of their lives, helps to recruit and retain top talent.”^13^

### Who

Particularly in large global companies such as Merck, determining what population is being served is important. Doing so allows initiatives to consider the unique demographic, cultural, educational, and other challenges faced by a specific population.

Merck already had several successful and sustained well-being efforts around the globe. However, Merck recognized the opportunity to do more. This initial focus was dedicated to understanding and improving the health status of the United States-based workforce through health promotion and prevention. However, the study team also knew that longer term, lasting improvements would require an appreciation of well-being that extends beyond physical and emotional health. Although starting with the United States-based workforce, the team was able to draw on best practices already discovered from other regions around the world, such as the successful ways to drive high employee engagement from Merck's Canadian corporate wellness programs.^14^

### What

Responding to the “what” allows the organization to describe succinctly what it is going to do. This also provides an opportunity to develop the required specific definitions and establish a general multiyear framework.

For the team, the “what is building a culture of health” looked like a deliberate and resourced effort to establish a workforce culture that promotes health, wellness, and safety and is focused on creating healthy daily habits.

The vision and scope of health and well-being were then further defined to encompass each of the 3 components: health, wellness, and safety.

- **Health** – Merck's mission is to improve health around the world. The company recognized the need to provide its own employees with a valuable suite of benefits to support their professional achievement and personal well-being.^15^ A healthy workforce helps enhance a competitive position in the marketplace. This is best accomplished by providing employees with programs and tools that promote health and prevention (at work and at home), address acute illness, manage chronic conditions, and support complex medical needs.^4^
- **Wellness** – The total wellness efforts recognize the need to reach beyond physical health to support emotional health and financial health, as well as safety. The Merck *LIVE IT* initiative had already been introduced in 2011. *LIVE IT* brings together health and wellness offerings under a single branded campaign. In many countries around the world, such as Brazil, Canada, China, India, the United Kingdom, and Spain, Merck employees have used *LIVE IT* as a foundation to build a local culture of well-being. In Canada, for example, the organization has already demonstrated improvements in biometrics, fitness, and emotional health, accompanied by fewer self-reported absences.^14^ Specifically, 1-year follow-up results of participants in the Canadian program included a −3.4 mm Hg decrease in systolic blood pressure, a 5 percentage point decrease in poor sleep, a 6 percentage point decrease in high emotional stress, and a 5 percentage point decrease in employees experiencing fatigue.^14^
- **Safety** – Merck already had a highly cultivated safety program in place. The vision for integrating safety into a culture of health demonstrated its commitment to redoubling efforts to build a culture of health that can complement and leverage a culture of safety. The value of safety in employee health and wellness has been well established with organizations such as the ACOEM publishing guidance on the importance of safety for a healthy workforce.^16^ The Global Safety and Environment team put processes and systems in place to bring them closer to achieving the vision of World Class Environmental, Health & Safety (EHS) performance. Consistent processes ensure that performance expectations are met and facilitate rapid sharing of proven practices globally. A training curriculum had already been established for EHS professionals at both management and employee levels. Additionally, audits, self-assessments, and inspections to find and fix deviations and identify opportunities to improve, were already part of safety programs. Developing parallel programs to apply to the culture of health and well-being logically follows.

### How

For the study team, the how started with a recognition that a sustainable culture of health requires strong leadership support, a grassroots champion network, a workplace environment that supports healthy decisions, and a strong and lasting commitment to marketing the value of being well within and across the organization. In short, leadership must champion health and wellness themselves before they can inspire others to do the same.

The decision was made to take a population health view and apply it to the workforce. This was complementary to the existing approach of providing employee benefits and services that support physical, emotional, and financial health. This view would require more than offering additional benefits and programs. Health and safety had to become part of the fabric of the workplace. All of this would require investment in people, time, and money. A best practice is to create a formal business case to clearly develop the reasons for, investment needed, expected returns, and risks associated with embarking on the initiative.

## Collecting Evidence to Build a Business Case for a Culture of Health and Well-Being

The team took a multistep approach to build a business case, starting with a search of the literature for supportive evidence to justify a commitment to building a culture of health, safety, and well-being. This search resulted in several key findings including:

- A healthy workforce is more productive and incurs fewer direct and indirect costs^17^
- There is a return on investment in health^18^
- Promotion of health and well-being results in greater employee engagement, trust, and satisfaction^19^
- A healthy workforce is a safer workforce – incurring fewer injuries^20^
- Publicly traded companies that are recognized for their commitment to building a lasting culture of health and well-being demonstrate superior stock market performance^9–11,21^
- By focusing on reducing the illness burden of the workforce it is possible to reduce both direct and indirect health care costs^22^
- There are benchmark organizations that have achieved corporate cultures of health and have established a road map or pathway for others who aspire to achieve the same result to follow^1^

Once enough evidence was gathered, focus was placed on what the organization had already achieved and this was compared to competitors and benchmark organizations. Several dozen large employers have been recognized for their benchmark culture of health efforts from organizations such as the National Business Group on Health (NBGH), ACOEM, KOOP Health Project, and HERO. The review confirmed that several health care manufacturers were ahead of Merck and were already award recipients and had published or presented in national forums.

This information gathering process was made somewhat easier when Merck applied for and was awarded a NBGH Best Employers for Healthy Lifestyles Silver Award in 2016.^23^ The process of applying for this award program included valuable feedback that helped identify strengths and opportunities. Additionally, in the process of deciding to participate in the NBGH Best Employers for Healthy Lifestyles Award program and identifying other award programs, a comprehensive list of industry standards that benchmark culture of health companies had achieved was developed. The standards required to achieve the CHAA from ACOEM^24^ and the requirements to obtain the KOOP Award from the Health Project^25^ were studied and documented as well. The CHAA emphasized the importance of integrating health protection with health promotion while the KOOP award highlighted the need to collect data demonstrating improvements in the illness burden of the workforce and covered lives over time. These references and checklists assisted the study team in detecting opportunities for improvement that shaped the strategy and tactics going forward.

Additionally, the team identified tools and references from the Centers for Disease Control and Prevention, the National Institute for Occupational Safety and Health, the World Health Organization, the Harvard School of Public Health, and the Integrated Benefits Institute (IBI). Particularly helpful were the following:

- The Whole Worker^26^
- SafeWell^27^
- WHO Healthy Workplace Framework and Model^28^
- Data modeling tools from the IBI Blueprint for Health^29^

The importance of aligning all potential contributors to a corporate culture of health, safety, and well-being was stressed by several key references.^26,30^ Following this guidance, the team took an initial inventory of all health- and well-being-related activities in the following areas or departments: human resources, environmental health and safety, occupational health, benefit administration, market research, marketing and communication, and global population health. The team also identified several health-promoting and safety programs to leverage and integrate. There were multiple touch points to promote employee health and well-being. The team collaborated across the enterprise to ensure the greatest impact.

The inventory exercise provided a set of existing programs that support a comprehensive health, safety, and well-being effort. Programs in industrial hygiene and workplace safety, occupational health, and the wellness programs under the *LIVE IT* brand, were identified as key programs to build on.

Providing a safe workplace for employees and contractors and complying with all applicable safety laws and regulations is of paramount importance. As a global biopharmaceutical company, employees have a vast array of work assignments—each with its own range of requirements. Work assignments may involve potential exposure to occupational hazards, such as noise, mixtures of chemicals, or hazardous biological compounds. Merck maintains a concerted effort to assess and control workplace hazards (chemical, biological, and physical) and to make sure that each employee's work assignment is safe and consistent with his or her evaluated capabilities.

Beyond industrial hygiene Merck already had robust workplace safety programs in place that aim to eliminate work-related injuries, illnesses, and unplanned events. Comprehensive safety programs that are part of the EHS management system focus on proper facility design, process controls, operation and maintenance procedures, protection systems, and emergency response capabilities. Much of the last 5 years had been spent working to establish a culture of safety with considerable success, reducing recordable injuries by 44% since 2012 (unpublished data; C. Gunther; February 2018). These programs include Target Zero, an initiative to drive a mind-set shift focused on helping people to make the right choices – to identify, eliminate, or control hazards, follow safe behaviors, and coach others. Target Zero includes training and tools to drive senior leader and employee engagement, along with behavior coaching and hazard recognition training. Sharing of safety incidents and near misses across the company are important components of the initiative. The practice of beginning meetings with a brief safety reminder, called a Safety Minute, also was initiated. This simple practice– the quick sharing of an incident, a near miss, or a safety tip as the first item on meeting agendas – has helped keep safety at top of mind and is commonly used to start meetings throughout the company. A more recent program, Safe by Choice, is an all-encompassing effort to drive a culture of safety in the manufacturing division. Safe by Choice emphasizes leader and employee roles and accountability in creating, maintaining, and promoting a safe work environment. The program reinforces key concepts through weekly web-based activities and monthly leadership videos, and by incorporating program messaging into standing weekly operations meetings.

Occupational health programs are developed and implemented in accordance with identified health risks and applicable regulatory requirements. Merck had invested in a global team of employee health professionals who were clinically trained and dedicated to preventing illness or injury from workplace hazards, as well as supporting efficient and effective quality health care for employees who become injured or ill. These professionals advise on and coordinate health care with providers or agencies to ensure a smooth treatment and recovery process, while complying with both company and applicable regulatory requirements. There also are on-site clinics at many of the larger sites. Although focused on occupational health, many of the on-site clinics offer employees routine health screenings, preventive services, and non–work-related acute episodic health care.

**FIG. 1.**
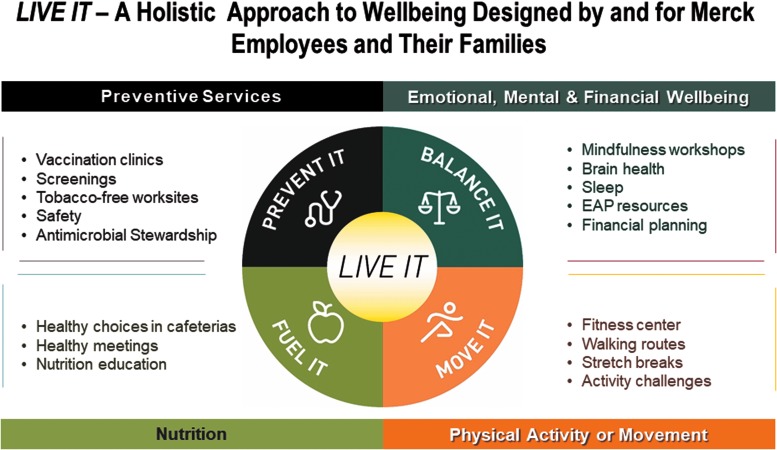
*LIVE IT* components. EAP, Employee Assistance Program.

Employee-focused health and wellness efforts, provided through global benefits, were branded under *LIVE IT*. Highlighted in Figure 1, *LIVE IT* comprises a variety of resources, tools, and services that support physical, emotional, financial health, and general well-being. *LIVE IT* initially was launched in the United States in 2011, and then throughout the global organization – across more than 26 countries, to more than 50,000 employees participating in some fashion, representing 75% of the global workforce – by 2017. This comprehensive program offers a foundation on which the team can further incorporate well-being as part of the fabric of everyday work life.

## Developing Baseline Metrics

To understand the future impact of a programmatic approach for wellness, it was important for the study team to assess the population's current state of health, the cost of illness to the organization, employees' understanding and engagement rates of programs as well as their perceptions regarding the company's current culture of health and well-being.

To accomplish this, the team identified 6 data sets to answer 3 important questions:

- What is the present state of workforce health?
- How much does the illness burden cost?
- How do employees feel about the programs and efforts deployed to improve health?

Using multiple data sources from health, disability, and pharmacy claims, health risk appraisals (HRAs), biometric screening, and employee engagement surveys, the team established a baseline assessment. The organization engaged a third-party data warehouse vendor that captured health claims data from medical, pharmacy, and personal health assessments. Data were prepared and measured using the same methodology described in the Canadian LIVE IT study and so will be described only briefly.^14^ De-identified aggregate data were used to better understand the illness burden and associated cost, as well as lifestyle risk factors for, active US employees, their spouses/partners, and dependents. These data were compared to benchmarks and used to identify areas worthy of addressing.

HRA and biometric data included sex, age, height, weight, personal and family history of cardiovascular disease and diabetes, smoking status, food choices, physical activity, access and use of preventive care, medication use, and blood pressure, as well measures of sleep quality, emotional stress, and depression. Medical and pharmacy claims data were analyzed to identify costs, diagnosed conditions, health care and prescription drug utilization, receipt of screenings, and preventive care such as mammograms and flu shots.

De-identified aggregate disability data were analyzed to understand the conditions causing short-term and long-term disability claims in the workforce and their impact on productivity. The IBI Total Costs of Workforce Health modeling tool set,^29^ which estimated the total costs of health care including absence, disability, performance, and productivity, also was deployed. The intent of using this data source was to estimate the cost impact related to lost time from presenteeism and lost productivity costs related to employee health issues.

Internal market research resources were used to survey employees about how well the company is meeting employees' health promotion needs as well as their awareness and utilization of current health promotion programs. Additionally, the company conducted a biannual employee engagement survey.

These analyses yielded 4 key findings. First, self-reported risks from the HRA such as poor diet, high stress, and being overweight are more prevalent than benchmark. Second, prevention measures could be improved, such as improving diet, physical activity, screenings, and vaccination coverage. Cancer screening rates exceeded industry averages but could be improved to meet Healthy People 2020 goals. Vaccination rates for flu and shingles were close to best practice but could be improved by setting vaccination goals and increasing awareness and engagement. Third was the increasing prevalence of prediabetes and diabetes in the aging employee cohort. Of the employees who completed an HRA, 37% were identified as at risk for diabetes because of being overweight, having a sedentary lifestyle, and eating an unhealthy diet. The fourth finding was that stress and depression impact employees' health and job satisfaction, and has an associated significant cost related to lost productivity. Of employees who rated themselves as having a poor level of health, 55% cited stress as the key reason.

Insights from the analysis led to the establishment of 2 strategic imperatives. The first was to improve health status in targeted areas, specifically cardiometabolic risk, screenings, vaccinations, and stress management. The second was to establish a workplace culture that promotes good health and prevention by focusing on daily habits. Programs that support workplace wellness included increasing physical activity/movement, nutrition, mindfulness, and safety. Additionally, the team recognized the need to increase awareness of the available *LIVE IT* resources.

## Developing Metrics and Goals to Measure Impact

With a baseline assessment established, the study team turned to identifying goals and relevant metrics to track progress toward best practice. For practical reasons, metrics that were easy to collect and trend were selected to be focused on to measure changes in performance against the 2 strategic imperatives. Goals and metrics under consideration include those shown in Table 1.

**Table 1.** Goals and Metrics

*Goal*	*Metric(s)*
Improve employee engagement	Internal biannual survey
Bend the benefits cost curve	Medical cost trend
Decrease injury and safety event rates	Occupational illness and injury trend
Maximize current investments in human capital	Absenteeism rates Disability rates Time to fill positions Turnover rates Retention of top talent Attraction of top talent
Achieve recognition in the field of health and wellbeing	Earn awards: National Business Group on Health's Best Employer for Healthy Lifestyle Award Everett Koop Corporate Achievement Award American College of Occupational and Environmental Medicine's Corporate Health Achievement Award American Heart Association's Workplace Health Achievement Index

## Building the Business Case

Using insights from the information gathered, the team developed a road map that outlined the actions, programs, and interventions that are expected to move the United States-based employee population forward in creating greater employee health and wellness. These plans include:

- Develop the well-being strategy and communications plan
- Increase appropriate adult vaccination coverage
- Encourage broader use of biometrics and cancer screenings
- Modify existing programs to address targeted conditions such as diabetes and cardiometabolic syndrome
- Increase opportunities to encourage physical well-being and enhance movement throughout the day
- Create tools, such as a “meeting tool kit” to raise awareness and encourage healthy behaviors
- Develop a *LIVE IT* champions network
- Expand tobacco-free campuses
- Provide training on stress-reducing techniques

A business case was then developed to increase awareness and obtain the resources needed to put these plans in place. The business case featured the present state of health, direct costs associated with employee and dependent health care, the impact of indirect costs on the organization, and an understanding of how employees feel about, and engage in the existing efforts to improve health. The business case provided insights to address the health-related gaps revealed in the analysis and, in parallel, proposed a strategy to evolve the corporate culture toward greater wellness. Based on evidence that correlates a culture of health with bending the health care cost curve, the business case addressed the potential financial benefits of such an effort as well as a proposed implementation time line and the resources needed. A concerted effort was made to provide data and metrics that would resonate with all corporate executives such as productivity, engagement, financial, risk management, reputation, and clinical factors.

Building the business case was a collaborative process, engaging employee health services, human resources, benefits expertise, external subject matter experts, market research, the commercial organization, safety, and the population health team. The study team met with several executives to gather input and guidance to ensure that different perspectives from across the organization had been considered. Finance was interested in the investment needed and the impact and timing on direct health care costs. Human resources sought to understand how this effort could fit with other corporate initiatives. Manufacturing and legal representatives recognized the connection to safety – and the association between health and injury rates.

Although there is still much work to be done, the business case presentation achieved alignment on 2 things: it was the “right thing to do” for employees, and this effort was consistent with the organization's corporate reputation. This was only the beginning. Now that resources have been secured, the hard work of putting plans in place will be ongoing and require diligent attention and focus on the overarching goal – to advance the culture of health and well-being at Merck.

## Lessons Learned: Guidance for Others

This article provides a summary of the beginning phases to augment a culture of well-being at Merck. However, it was not without challenges. First, comprehending the health and wellness status of employees from different departments and different locations (even around the globe) required amassing diverse data sets and identifying information gaps. In this case, the team developed and conducted an additional survey to appreciate how employees felt about the benefits that were offered – questions that had not been addressed previously. Each company has its own unique set of data from which to gain insights – ranging from simple surveys to an amalgamation of personal health assessments, medical and prescription drug claims, and disability data. Second, the business case for investing in well-being requires an organized campaign and navigation across leaders. Garnering support from the many functional areas could happen only after understanding the divisional priorities, needs, and wants that could be addressed through such an effort. Leaders have different interests and perspectives. Employee engagement and talent acquisition may be critical to some, while for others, manufacturing productivity, safety, or cost considerations are essential. A solid business case speaks to each leader – with evidence from the literature, a hypothesis, and the anticipated benefits to the company. A common misstep is believing that the business case is “one size fits all.” Lastly, the always present competing priorities – for investments, time, and human resources. Timing the presentation in the natural ebb and flow of business issues is important. However, more critical to success is ensuring that management appreciates that building a culture of well-being is a marathon. It requires adequate and sustained investments to ensure lasting benefit.

Delivering on the promise of a culture of health demands a unified effort from a broad array of leaders and managers across the company. Even with the initial successes, competing for limited resources will be an ongoing challenge. The team believes and recommends to others that recording evidence of early impacts will be important to reinforce and support a budget. The team's experience suggests to others to measure progress as best they can. Large companies should invest and leverage an integrated data warehouse to draw needed insights and record improvements. Smaller organizations can track participation rates and program completion rates, as well as satisfaction and engagement levels. Investment in building a culture of health can be justified simply by moving these dials.

## Conclusions and Next Steps

There is increasing evidence that a healthy and safe workforce can provide a competitive business advantage. This article provides a blueprint designed by a large employer to gain awareness and support for building a culture of health and well-being. Starting with a comprehensive and data-rich analysis of the current state of employee health and culture, a small team established the business case, aligned strategic partners, created an implementation plan, and engaged the C-suite to garner support and resources.

**FIG. 2.**
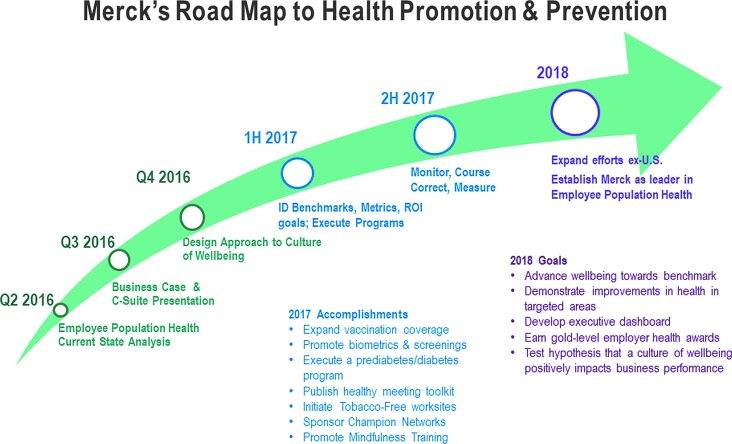
Road map to health promotion and prevention. H, half; Q, quarter; ROI, return on investment.

Internal discussion within this employer raised awareness of the opportunity and reinforced the need for it to advance employee health and well-being. Approval was received to add additional staff dedicated to executing health promotion and prevention initiatives. Figure 2 displays important tollgates identified by this employer in its strategic plan through 2018. These include selection of key metrics and goals, establishment of a monitoring process to assure that the programs stay on course, and expansion of programs beyond the United States.

Employers have much to gain by building cultures of health and well-being. Leadership support is an integral part of success, as are grassroots efforts to promote healthy and safe behaviors in the workplace. An integrated approach that connects efforts by human resources, occupational health, environmental health and safety, and cross-functional leadership can multiply the impact.

The primary limitation of this case study is that it is early stage, and thus, the results on the impact on employee health are minimal. However, the intention is to continue this line of research and report on results, both positive and negative, as they are available. The team also acknowledges that not all companies have the resources available to very large employers such as the one featured here. That does not mean that smaller organizations cannot develop a robust and sustainable culture of health. They simply must find the right-sized solutions for their workplace.

In conclusion, the work outlined here is just the beginning as a large employer embarks on the journey to improving its culture of employee health and well-being. This work is ongoing and requires forethought and a commitment to developing and following a multiyear road map as well as engaging in continuous evaluation. The goal is to discover and implement best practices that fit a given workplace environment. One size does not fit all in this endeavor, but it is the team's hope that this early experience can assist and encourage other employers to take the first steps in a similar pursuit.
